# Retraction: Digital economy, resource distortion and low-carbon inclusive development-Evidence from the perspectives of a threshold effect and knowledge spillover effect

**DOI:** 10.1371/journal.pone.0353312

**Published:** 2026-07-08

**Authors:** The PLOS One Editors

The *PLOS One* Editors retract this article [1] due to concerns about:

- Compliance with PLOS policy on Authorship.
- Extensive overlapping text in the introduction, and section 4.2., with a previously published article [2] that was not cited or declared to PLOS as related at the time of submission.

Additional concerns were identified about compliance with PLOS policy on Artificial Intelligence Tools and Technologies and the peer review process. The Editors have no evidence of any author involvement in the peer review concerns.

Corresponding author FD replied to acknowledge that the two articles share the same author group and address a closely related research theme, and stated that the authors regret any unintended lack of citation to the prior work. The Editors do not consider this matter to be resolved.

Corresponding author FD declared that the authors used AI-assisted tools to improve the readability of their manuscript.

FD and FW did not agree with the retraction. GY, ZM, XW, and FZ either did not respond directly or could not be reached.
